# Identification and Validation of Novel Hedgehog-Responsive Enhancers Predicted by Computational Analysis of Ci/Gli Binding Site Density

**DOI:** 10.1371/journal.pone.0145225

**Published:** 2015-12-28

**Authors:** Katherine Gurdziel, David S. Lorberbaum, Aaron M. Udager, Jane Y. Song, Neil Richards, David S. Parker, Lisa A. Johnson, Benjamin L. Allen, Scott Barolo, Deborah L. Gumucio

**Affiliations:** 1 Department of Cell and Developmental Biology, The University of Michigan, Ann Arbor, MI 48109, United States of America; 2 Department of Computational Medicine and Bioinformatics, The University of Michigan, Ann Arbor, MI 48109, United States of America; 3 Cellular and Molecular Biology Program, The University of Michigan, Ann Arbor, MI 48109, United States of America; University of Iceland, ICELAND

## Abstract

The Hedgehog (Hh) signaling pathway directs a multitude of cellular responses during embryogenesis and adult tissue homeostasis. Stimulation of the pathway results in activation of Hh target genes by the transcription factor Ci/Gli, which binds to specific motifs in genomic enhancers. In *Drosophila*, only a few enhancers (*patched*, *decapentaplegic*, *wingless*, *stripe*, *knot*, *hairy*, *orthodenticle*) have been shown by *in vivo* functional assays to depend on direct Ci/Gli regulation. All but one (*orthodenticle*) contain more than one Ci/Gli site, prompting us to directly test whether homotypic clustering of Ci/Gli binding sites is sufficient to define a Hh-regulated enhancer. We therefore developed a computational algorithm to identify Ci/Gli clusters that are enriched over random expectation, within a given region of the genome. Candidate genomic regions containing Ci/Gli clusters were functionally tested in chicken neural tube electroporation assays and in transgenic flies. Of the 22 Ci/Gli clusters tested, seven novel enhancers (and the previously known *patched* enhancer) were identified as Hh-responsive and Ci/Gli-dependent in one or both of these assays, including: *Cuticular protein 100A* (*Cpr100A*); *invected* (*inv*), which encodes an *engrailed*-related transcription factor expressed at the anterior/posterior wing disc boundary; *roadkill (rdx)*, the fly homolog of vertebrate *Spop*; the segment polarity gene *gooseberry (gsb)*; and two previously untested regions of the Hh receptor-encoding *patched (ptc)* gene. We conclude that homotypic Ci/Gli clustering is not sufficient information to ensure Hh-responsiveness; however, it can provide a clue for enhancer recognition within putative Hedgehog target gene loci.

## Introduction

The Hedgehog (Hh) signaling pathway plays multiple roles in embryonic organ development and adult tissue homeostasis across animal phyla [1,2,3]. Hh signaling directs specific cell fate choices, controls tissue patterning and governs cell proliferation. Several human developmental diseases are caused by altered Hh signaling, including spina bifida, exencephaly [4], holoprosencephaly [5], cleft lip/palate [6], and a host of malformations in vertebral, anal, cardiac, tracheal, esophageal, renal, and limb tissues (together known as VACTERL Association;[7]). Aberrant Hh signaling is also responsible for several cancers, including basal cell carcinoma, medulloblastoma and rhabdomyosarcoma [8]. Recently, cancers of the pancreas, colon, ovary, stomach and lung have also been associated with increased Hh signaling [8,9], prompting initiation of clinical trials with Hh antagonists for some of these conditions [10,11,12,13].

The Hh-regulated Gli family transcription factors (including Cubitus interruptus (Ci) in the fly and Gli1-3 in mammals) are highly conserved across metazoans, as is the sequence of the preferred consensus Ci/Gli binding site [14,15]. Despite the functional importance and high conservation of the Hh pathway, surprisingly little is known about its target genes in any organism. These target genes and their associated enhancers, which are responsible for the genomic response to Hh in development and disease, have significant potential therapeutic and diagnostic value.

One method for identifying putative enhancers is chromatin immunoprecipitation (ChIP) [16,17,18,19,20], though such data are subject to the spatiotemporal limitations of the analyzed cells or tissues and can be diluted by a high number of false positive binding sites. While many potential murine Hedgehog-responsive enhancers have been pinpointed in this manner, relatively few have been functionally verified by mutagenesis of transcription factor binding sites [17,18,19,20]. In *Drosophila*, an alternative approach, DamID which fuses a DNA interacting protein to DNA adenine methyltransferase leading to methylation near binding locations, identified 52 potential Ci/Gli target enhancers, though none of these were functionally verified by mutagenesis of Ci/Gli sites [16]. To date, only seven *Drosophila* enhancers have been shown by mutational analysis to be Ci/Gli-dependent [21,22,23,24,25,26,27], which limits our understanding of the basic rules that govern their activity and context specificity.

Analysis of the known *Drosophila* Hh enhancers reveals that three (regulating *ptc*, *wg*, and *knot*) contain clusters of three or more Ci/Gli binding sites, while the remaining enhancers (of the *dpp*, *stripe* and *hairy* genes) contain two sites [21,22,23,24,25,26,27]. These examples, and findings in other systems [28,29,30,31,32] suggest that homotypic clustering might be a relevant indicator of Hh enhancer activity in the fly. To test this, we computationally identified regions of the fly genome in which the density of Ci/Gli binding sites is enriched relative to chance expectation. We then tested the ability of these regions to: 1) drive Hh-dependent activity in the developing chicken neural tube, and 2) direct tissue-specific gene expression in a *Drosophila* transgenic reporter model. Importantly, the functional significance of the Ci/Gli binding motifs was also tested by mutation of these sites within each active enhancer. Of the 17 top clusters, four (23%) drove reporter expression in a known Hh domain and/or in a Ci/Gli-dependent fashion in one or both assays. Thus, while some Hh-regulated enhancers indeed contain homotypic clusters of Ci/Gli motifs, not all such clusters function as enhancers *in vivo*.

We also asked whether Ci/Gli site clustering could be used to predict the location of enhancers in genes that are known or putative targets of Hh signaling. We identified five such Ci/Gli site clusters, four of which were subsequently validated as Hh enhancers by functional assays (80%). Thus, altogether, our analysis of clustered Ci/Gli sites identified eight Hh enhancers, including seven novel enhancers and one previously identified *ptc* enhancer. These findings double the number of functionally verified Hh enhancers.

## Materials and Methods

### Computing resources

Except where otherwise indicated, all computational steps were performed using custom Perl scripts, which are available for download at https://github.com/um-gurdziel/GurdzielUdagerLorberbaum2015. Overlap between coordinates in bed file format were performed using the UCSC Table Browser.

### Definition of putative Ci/Gli binding sites

A mono-nucleotide distribution matrix for Ci binding sites, derived from *in vitro* competitive DNA binding assays with recombinant Ci protein and labeled oligonucleotides, was obtained via the Genomatix Software Suite (www.genomatix.de; Genomatix, Germany) [14]. The consensus index vector for such a matrix reflects the degree of nucleotide preference at each position; values range from 0, indicating equal preference for any of the four nucleotides, to 100, indicating strict preference for a single nucleotide [33]. The matrix similarity score (MSS) for a given site is calculated as the ratio of its matrix-vector product to that of the consensus site, as described previously [33], and MSS values range from 0 to 1 (where 1 equals an exact match to the consensus site). The first nine of the eleven positions in the Ci matrix have consensus index vector values greater than 70, suggesting that they contain a high degree of specific information about potential Ci binding. Thus, these matrix positions were used to define a set of 211 9-mers (422 in sense and antisense directions) that pass a minimum level (0.75) of overall matrix similarity (i.e. with a MSS ≥ 0.75) to the optimal consensus Ci site (GACCACCCA) (S1 Table) [14,33] and also contain concordant (C and C or G and G) nucleotides in the 4^th^ and 6^th^ position, which are critical for Ci binding [15].

### Identification and annotation of predicted Ci binding sites in genomic sequence

Genomic sequence files (chromFa) for *D*. *melanogaster* (Dm) and *D*. *pseudoobscura* (Dp) were downloaded from UCSC Genome browser (genome.ucsc.edu) build dm3 [34,35,36]. The genomic coordinates of predicted Ci/Gli binding sites were identified for chr2R, chr2L, chr3R, chr3L, chr4, and chrX (build dm3); and chr2, chr3, chr4 and chrX (build dp3). Each putative Ci/Gli binding site was annotated for nearest gene/transcript, distance to nearest gene/transcript, and associated gene/transcript feature transcript using refFlat files obtained from UCSC Genome Bioinformatics. Ci/Gli clusters were defined as regions containing at least three and at most ten putative Ci/Gli binding sites within a maximum distance of 1000 base pairs (bp) (measured from the outside ends of the flanking sites [36,37]. Predicted sites were also annotated with respect to the nearest CTCF boundary region [38]. Cluster regions that contained predicted Ci binding sites that mapped to exons or repeat regions were excluded. Repeat regions often have regulatory function [39,40]. However, testing the regulatory activity of Ci binding motifs in repetitive sequences, and the effect of their clustering in these regions, was beyond the scope of this study.

### Background Modeling

To identify regions of the genome that exhibit a higher density of Ci/Gli sites than would be expected by chance, we compared the actual distribution of Ci/Gli sites to a randomized background model. Three different modes of background modeling were examined. For Model 1 (Random), all bases in the genome were randomized, as was done in a previous analysis of clustered binding sites for Suppressor of Hairless [32]. For Model 2 (Shuffle 3mer), the genome was parsed into contiguous 3-mers and these were then shuffled to create the background. In Model 3 (Flip GC/AT), each base was randomly flipped between itself and its complementary base pair (e.g., G will randomly become G or C; A will become A or T; C will become C or G; T will become T or A). On the basis of the data shown in Results (S1 Fig), only the Flip GC/AT model generates background genomes that most closely represent the GC content surrounding Ci/Gli sites in the native genome. Since GC rich Ci/Gli sites will occur by chance more often in GC rich than AT rich regions, use of a randomization model that homogenizes the AT/GC landscape would artificially reduce the density of expected Ci/Gli sites in GC rich areas and increase this density in AT rich regions. Therefore, using the Flip GC/AT strategy, background models were generated separately for the Dm and Dp genomes for comparison to each native genome.

### Generation of artificial genomic sequence and random genomic distributions of binding sites

On a chromosome-by-chromosome basis, 1000 sets of background genomic sequences were generated using the Flip GC/AT method. However, base flipping resulted in fewer Ci/Gli sites in the randomized chromosomes, relative to the native Dm or Dp genome. To correct for this, putative Ci/Gli binding sites were identified in each of the 1000 background genomic sequences and the genomic coordinates of each site was recorded. Site motifs, tagged with their location coordinates, were pooled into a master list of possible site positions. This master list was used to re-create 100 background chromosomes for each chromosome, such that each background chromosome contained the same composition of Ci/Gli sites (overall number and motif) as the native Dm or Dp chromosome (see Results).

### Assessment of relative Ci/Gli binding site clustering

Ci/Gli site clusters were defined as regions containing at least three and at most ten putative Ci/Gli sites within a maximum distance of 1000 base pairs (bp; measured from the outside ends of the flanking sites). The genomic coordinates of each cluster were cataloged, and clusters were subsequently filtered for the presence of at least one predicted binding site with a MSS ≥0.81. This was done to decrease the number of clusters comprised entirely of low scoring sites, substantial portions of which are predicted to be non-functional. Clusters that contained exon or repeat elements were excluded. Clusters for which the Ci/Gli binding sites themselves accounted for more than 25% of the end-to-end cluster length were also excluded, since the majority of such clusters were composed of repetitive sequence. For each cluster, the number of binding sites expected to be present by chance for that specific genomic region was determined from 100 control reconstructed genomes as described in Results. A clustering coefficient (CC) was defined as the number of Ci/Gli sites observed in a given interval of the native genome (at a given location) divided by the average number of Ci/Gli sites in the same region of the background genome (at the same location). To enrich for clusters likely to represent enhancers, we selected a CC cutoff of four which captured all of the previously known clustered Hh enhancers. Importantly, the CC score was used as a filter, and not as a ranking tool.

### Orthologous enrichment of Ci clusters

Clusters were identified and annotated in the Dp genome exactly as described above for Dm. Background modeling for the Dp genome was done by Flip GC/AT; 1000 randomized genomes were generated and corrected as outlined above for number and affinity class to make 100 randomized, corrected Dp genomes for comparison to the native Dp genome. Clusters identified in the Dp genome were selected according to the same criteria as for the Dm genome (cluster size ≤ 1000; 3–10 Ci/Gli sites; CC ≥ 4; at least one site with MSS ≥ 0.81). The coordinates for enriched clusters of Ci/Gli binding sites (CC ≥ 4) were determined for Dm and Dp and compared using the LiftOver tool available from UCSC Genome Bioinformatics [36]. All clusters that were present in orthologous positions of the Dm and Dp genomes (i.e., with an overlap of one or more bases, irrespective of sequence identity) were selected for further analysis.

### Cloning of putative enhancer regions for testing

Putative enhancer regions in the Dm genome were visualized in the UCSC Genome Browser, and using the Conservation track (12 Flies, Mosquito, Honeybee, Beetle Multiz Alignments & phastCons Scores), the ends of an individual enhancer element were extended to include contiguous highly conserved sequence [41]. Putative enhancers were amplified from *w* ^*1118*^ genomic DNA using template-specific PCR primers (S2 Table). A CACC extension was added to the end of one primer to facilitate directional cloning. PCR fragments were cloned into the pENTR/D-TOPO vector using the standard kit (Invitrogen) and then shuttled into either Ganesh-G2 [42] or HP-desteGFP [43] vectors using the Gateway^®^ cloning system (Invitrogen). Ci binding site mutations (C4A) were introduced by overlap extension PCR, as previously described [44]. QuikChange mutagenesis (Stratagene) was also used to mutate some Ci binding sites. pCIT was generated by replacing eGFP in pCIG [45] with TdTOMATO, which was cloned into the location between the third PmlI site and the NotI site in pCIG. *SmoM2*-pCIT was generated by cloning rat *SmoM2* into the XhoI and ClaI sites of pCIT.

### *Drosophila* transgenesis

Transformation was achieved by injection of *w* ^*1118*^ or *ZH-attP-86Fb* embryos, essentially as described previously [46,47]. A current protocol is available at: http://sitemaker.umich.edu/barolo/injection. For *w* ^*1118*^ transgenesis, at least three independent lines were examined; one or more lines were examined for *ZH-attP-86Fb* transgenesis.

### *Drosophila* tissue analysis

Since Hh is active in a variety of tissue contexts in the embryo (brain, gut, muscle, segmental stripes etc.), we utilized embryos at stages 9–13 to gain an unbiased view of all of these contexts. Additionally, we specifically examined the wing imaginal disc since this is a well-known and well-characterized expression domain for Hh signaling. Of the 22 genes selected for analysis (Table 1), 17 are expressed in the embryo or imaginal disc [48,49,50]. There are no data on two (CG5475, CG4704) and three others (beat-IV, BDGP, HGTX) are not reported to be expressed in these sites, but these have been incompletely studied. For imaginal disc analysis, 3rd instar wandering larvae were collected from vials, and discs were dissected fresh and fixed in 4% paraformaldehyde. For embryo analysis, embryos were collected in 6-hour batches at 25°C, dechorionated in 100% bleach, fixed in 4% paraformaldehyde, and devitellinized by shaking in methanol and heptane.

**Table 1:** Assessment of Hh response.

Annotated Gene	Genomic coordinates (dm3)	Number of Ci/Gli Sites	Average MSS	Hh Responsive in Chicken Neural Tube	Hh Responsive in Transgenic Fly
*ptc* ^*-0*.*6*^	chr2R:4536264–4536572	3	1.000	+	+
*inv* ^*+16*.*8*^	chr2R:7378801–7380000	4	0.941	+	-
*Sox100B*	chr3R:26894840–26896225	3	0.920	-	-
*inv* ^*+18*.*6*^	chr2R:7380576–7381900	4	0.903	+	+
*beat-IV*	chr3R:19385801–19387033	5	0.899	-	-
*CG6475*	chr3R:17227902–17229095	4	0.898	-	-
*CG34139*	chr3R:16067525–16068300	3	0.893	-	-
*Plc21C*	chr2L:308225–309200	4	0.892	-	-
*CG4704*	chr3R:18671231–18671930	3	0.891	-	-
*Bi*	chrX:4316001–4317440	4	0.886	-	-
*HGTX*	chr3L:14583895–14584670	4	0.886	-	-
*Cpr100A*	chr3R:26692110–26692580	3	0.886	+	-
*Ets21C*	chr2L:550010–551035	4	0.885	-	-
*CG12541*	chrX:6927600–6928375	5	0.884	-	-
*Sp1*	chrX:9613671–9614922	4	0.881	-	-
*Hth*	chr3R:6433650–6434996	5	0.879	-	-
*Ko*	chr3L:21072420–21073658	3	0.879	-	-
*ptc* ^*+5*.*3*^	chr2R:4542467–4545417	7	0.875	-	+
*ptc* ^*-2*.*8*^	chr2R:4531601–4534319	5	0.847	-	+
*Rx*	chr2R:16820211–16822050	5	0.845	-	-
*Rdx*	chr3R:9815295–9817061	3	0.838	+	+
*Gsb*	chr2R:20952400–20953750	7	0.834	-	+

The chicken neural tube (CNT) and transgenic fly (TF) assays together identified eight predicted regions as Hh-responsive. Both assays showed positive Hh activity for *inv* ^*+18*.*6*^ and *rdx* as well as the previously identified *ptc* enhancer region. Five additional regions were positive in only one assay: CNT: *Cpr100A* and *inv* ^*+16*.*8*^; TF: *gsb* and two *ptc* additional genomic regions (*ptc* ^*+5*.*3*^ and *ptc* ^*-2*.*8*^). All enhancer regions were verified by mutagenesis to be Ci/Gli binding site dependent.

### Chicken in ovo electroporations

Chicken neural tube electroporations were performed essentially as described previously [51]. Briefly, 500 ng/μl of reporter vector and 500 ng/μl of either pCIT or *SmoM2*-pCIT was dissolved in PBS with 50 ng/μl of Fast Green and injected into the neural tubes of Hamburger-Hamilton stage 10–12 chicken embryos. Approximately 48 hours following electroporation embryos were recovered and fixed in 4% paraformaldehyde for subsequent immunofluorescent analysis. Fertile eggs were obtained from the Michigan State University Poultry Farm.

### Immunofluorescence and microscopy

*Drosophila* embryos and imaginal discs were blocked with 10% BSA in phosphate-buffered saline (PBS) with 0.1% Triton X-100. The following primary antibodies were used overnight at 4°C: rabbit anti-GFP IgG antibody (1:200; Life Technologies A11122), mouse anti-Ptc (1:50, DSHB; APA1) and mouse anti-En (1:50, DSHB; 4D9). Samples were then incubated in the following secondary antibodies for 2 hours at room temperatures, Alexa Fluor 488-conjugated goat anti-rabbit IgG antibody (1:2,000; Life Technologies A11008) and/or Alexa Fluor 468-conjugated goat anti-mouse IgG antibody (1:2,000; Life Technologies A11004). Embryos and imaginal discs were mounted on glass slides using ProLong Gold with DAPI and imaged on an Olympus BX-51 upright microscope, Nikon A1 confocal with Ti-E microscope or Olympus FluoView 500 Laser Scanning Confocal Microscope. For direct comparisons, wild type and mutant constructs were processed in parallel including being imaged on the same day, using the same exposure settings.

Immunofluorescent analyses of chicken neural tubes were performed essentially as described previously [52]. The antibodies used were as follows: 1:20 Mouse IgG1 anti-NKX6.1 (DSHB; F55A10). DAPI (Life Technologies) was used at a dilution of 1:30,000. All secondary antibodies (Alexa Fluor; Life Technologies) were used at a dilution of 1:500. Primary antibodies were incubated overnight at 4°C, followed by incubation with secondary antibodies for one hour at room temperature. Images were collected with a Leica SP5X confocal microscope.

## Results

### Computational identification of clustered Ci/Gli sites across the *Drosophila* genome

To test if clustering of Ci/Gli sites could be used to predicted Hh enhancers, we developed a computational strategy to identify all regions of the genome that contain clusters of 3–10 Ci/Gli sites that are enriched above chance expectation. Since the Ci/Gli binding sequence is highly GC rich, these sites are more likely to occur by chance in GC rich regions of the genome. Thus, to achieve an unbiased assessment of clustering likelihood, it was important to utilize a background model with a GC landscape similar to that of the native genome. Three different background models were examined (see Materials and Methods for details). The three models were compared by mapping all predicted Ci/Gli sites (MSS ≥0.75) and examining the GC content of the genomic sequence surrounding each predicted Ci/Gli site (S1 Fig). Importantly, the randomized (Model 1) and shuffled 3-mer (Model 2) strategies significantly change (i.e., homogenize) the GC context around Ci/Gli sites, while the Flip GC/AT model (Model 3), by its nature, faithfully replicates the GC context of Ci/Gli sites in the real genome; thus, this model was selected for use.

An accurate assessment of the relative density of Ci/Gli clusters found in the native genome also requires that the background genomes contain a similar composition (number and type) of Ci sites as the native genome. After generating background genomes using the Flip GC/AT method, we noticed that the total number of predicted Ci/Gli binding sites on each chromosome was consistently reduced compared to the native Dm genome (S2A Fig). Left uncorrected, this deficit in total sites would lead to an artificial enrichment of clusters of Ci/Gli sites in the Dm genome when compared to the background model. To correct for this discrepancy, we re-built background chromosomes (see detail in Materials and Methods) so that they contained the same number of each type of Ci/Gli binding site (based on matrix similarity score) found in the Dm genome (S2B Fig). Relative enrichment of Ci/Gli clusters in each genomic region was then assessed across the native genome by direct comparison to the 100 rebuilt background chromosomes (S2C Fig).

### Ci/Gli cluster analysis in *Drosophila melanogaster*

**Fig 1:**
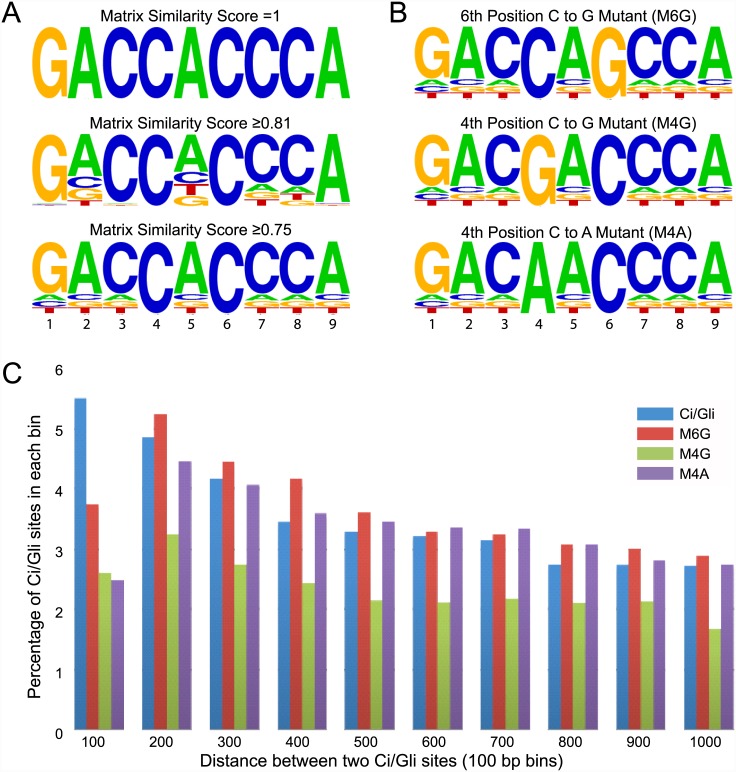
Pipeline for detection and validation of Hh-responsive enhancers. Ci/Gli cluster identification and background genome generation were performed as outlined in S2 Fig. The cluster (CC) for a given genomic region was calculated as the total number of sites observed in the Dm or Dp genome (observed) divided by the average number of sites per background genome for that species (expected). Clusters of Ci/Gli sites with a (CC) ≥ 4 were further filtered as follows: a) Clusters were required to contain at least one Ci/Gli site of ≥0.81 MSS; b) Dm Clusters were required to overlap in position (but not sequence) with a cluster in Dp; c) Clusters in exon or repeat regions were excluded. The entire table of selected clusters, sorted by chromosomal location, is provided in S4 Table. The list of clusters was then ranked by average MSS of the predicted Ci/Gli sites and the top 17 were examined functionally (these included 16 novel hits and one known enhancer, *ptc* ^*-0*.*6*^). The Hh-responsive enhancer activity of genomic regions containing selected clusters was functionally evaluated by means of a transgenic fly assay as well as by chicken neural tube electroporation. For genomic regions that showed apparent Hh responsiveness, Ci/Gli sites were mutated and re-assayed to confirm direct Ci/Gli regulation.

The complete pipeline for identification of enriched clusters of Ci/Gli sites and examination of their potential as Hh enhancers is provided in Fig 1. Clusters of 3–10 Ci/Gli sites (maximum end-to-end distance 1000 bp) were identified in the native Dm and Dp genomes. Background modeling and background correction was performed separately for Dm and Dp. For each putative cluster, a cluster coefficient (CC) was defined as the number of Ci/Gli sites in a given genomic region divided by the average number of Ci/Gli sites in the same genomic location in 100 control genomes (schematically illustrated in S2C Fig). Only clusters with a CC of greater than or equal to 4 and at least one Ci/Gli site with a MSS of 0.81 or greater were chosen for subsequent analysis. These filters (1kb length; CC ≥ 4; one site ≥ MSS of 0.81) were designed to increase the likelihood that functional enhancers would be identified. As an additional stringency filter, we required that Ci/Gli site clusters be present in orthologous regions of both Dp and Dm genomes (see Materials and Methods for details). S4 Table lists all selected Dm clusters with a CC greater than or equal to 4 (ranked by order of Ci/Gli site density and average MSS). We sorted these results by average MSS (high to low), to strengthen the likelihood that all of the Ci/Gli sites located within any putative cluster were capable of binding Ci/Gli, and observed that sites in a known Hh-regulated enhancer of the *ptc* gene [21] had the maximum average MSS of 1 (S4 Table). In addition to this known enhancer region, we selected the next 16 putative Hh enhancer regions for functional validation (Table 1).

### Functional verification of Ci/Gli-driven enhancers in a chicken neural tube assay

We first screened for possible enhancer function of the 16 novel genomic regions (Table 1) in the developing chicken neural tube, one of the best-studied sites of Hh signaling [53]. In this assay, Hamburger-Hamilton stage 11 embryos are electroporated with DNA reporter constructs in which the putative enhancer is cloned upstream of a minimal promoter driving EGFP expression (see Materials and Methods). This assay has been previously used to validate enhancers for multiple signaling pathways [20,54,55,56,57,58,59,60]. Endogenous Sonic Hedgehog (SHH) produced by the notochord and floorplate drives expression of Hh-dependent enhancers in the ventral half of the neural tube [53]. Additionally, to further increase the sensitivity of our assay, we co-electroporated a constitutively active form of *Smoothened* (*SmoM2*) [61], which activates Hh signaling throughout the neural tube. Successful activation of Hh signaling by *SmoM2* is readily detectable as an expansion of the expression domain of the known Hh target gene, NKX6.1 [20,55], on the electroporated side of the neural tube. An RFP-expressing plasmid (pCIT) was co-electroporated to confirm the success of the electroporation. For those enhancers that demonstrated apparent Hh activation (expression of the enhancer-containing construct, but not the enhancer-less construct, in the presence of *SmoM2*), Ci/Gli-dependent activity was further confirmed by mutagenesis of the Ci/Gli binding sites.

**Fig 2:**
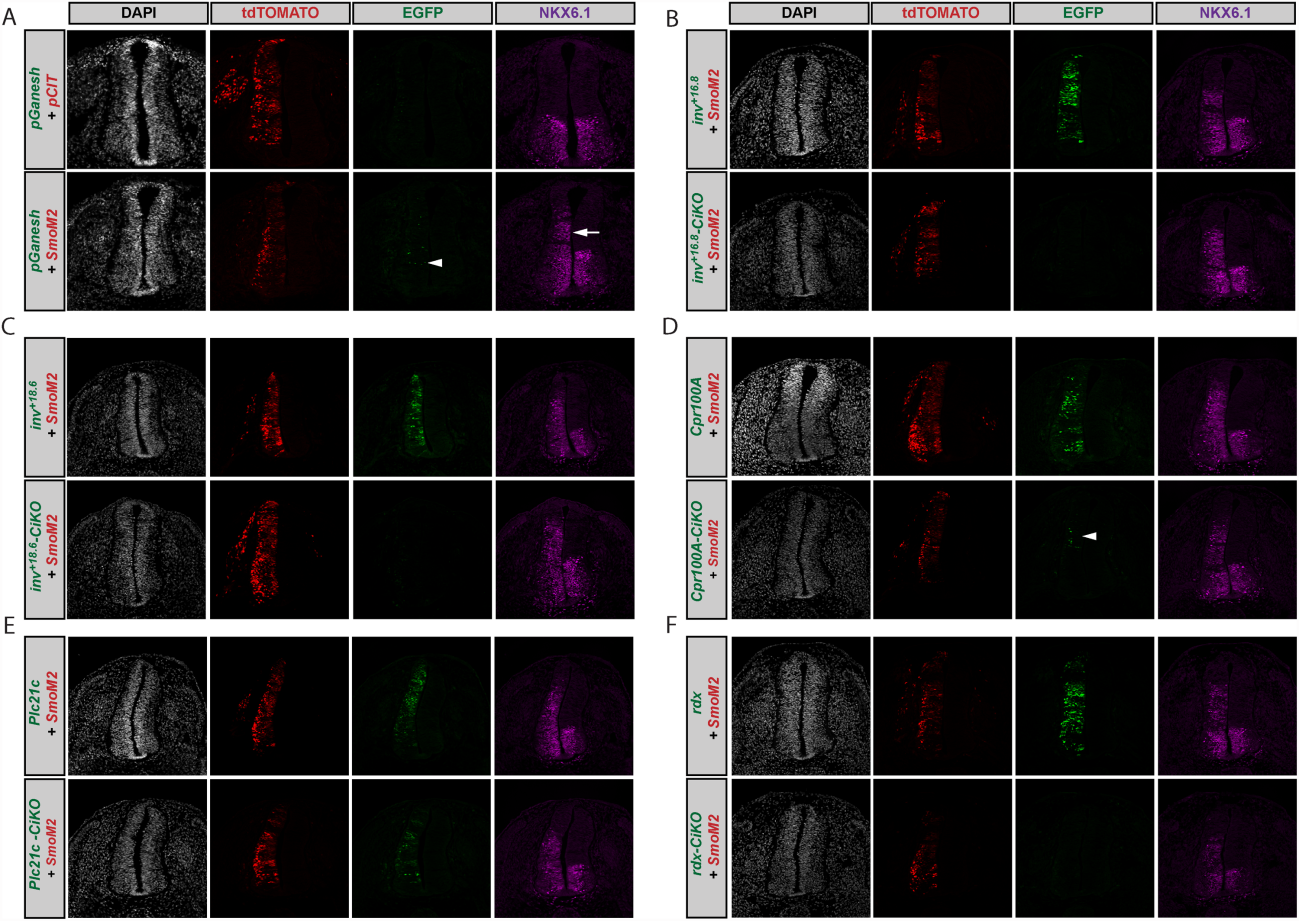
Validation of predicted Hh-responsive enhancers in the chicken neural tube. Transverse sections of Hamburger-Hamilton stage 21–22 chicken embryos are shown. DAPI (grayscale, far left column) depicts nuclei. tdTOMATO (red, middle left column) marks cells electroporated with pCIT or *SmoM2*. GFP (green, middle right column) reports enhancer activation. Anti-NKX6.1 antibody staining (magenta, far right column) denotes Hh-responsive cells. (A) Chicken embryos co-electroporated with an enhancerless pGanesh construct (containing only an Hsp70 minimal promoter) and either pCIT or a constitutively active *SmoM2*. An arrowhead (middle right column; bottom row) depicts a few GFP positive cells in pGanesh electroporated embryos. Note the ectopic NKX6.1 expression (far right column) indicative of overactive Hh signaling in electroporated cells (white arrow). (B-E) Candidate Hh-responsive *inv* ^*+16*.*8*^ (B top row), *inv* ^*+18*.*6*^ (C top row), *Cpr100A* (D top row), and *Plc21C* (E top row) constructs all exhibit GFP expression in cells in which Hh is activated by co-electroporation of *SmoM2*. However, chicken embryos co-electroporated with *SmoM2* in combination with a Ci/Gli-binding deficient mutant (CiKO) of each candidate (bottom rows) show a complete absence of GFP expression in the case of *inv* ^*+16*.*8*^ *-CiKO* (B) and *inv* ^*+18*.*6*^ *-CiKO* (C), despite ectopic NKX6.1 expression in both conditions (far right column). *Cpr100A-CiKO* (D) has a greatly diminished expression pattern with only a few GFP positive cells (white arrowhead) remaining (middle right column; bottom row). *Plc21C-CiKO* (E) does not show loss of GFP expression, indicating that it is not a direct Hh target, since its response to Hh signaling is not Ci/Gli dependent. *Rdx* (F top row) GFP expression corresponds to Hh expressing cells and shows no expression once Ci/Gli sites are mutated (*rdx-CiKO* bottom row).

Of the 16 computationally predicted enhancers tested in this way, four drove Hh-enhancer dependent expression in the chicken neural tube assay (Fig 2). An intronic sequence of the *invected* (*inv*) gene harbors two of these active regions, each containing a cluster of four Ci/Gli sites with MSS ≥0.81. Both regions drive expression in the presence of co-electroporated *SmoM2* and mutagenesis of the Ci/Gli binding sites abrogates this response in both cases (Fig 2B and 2C).

Two additional predicted enhancers, located near the genes *Cpr100A* and *Plc21C*, also showed expression in the chicken neural tube assay (Fig 2D and 2E). However, mutation of the Ci/Gli sites abrogated EGFP expression only in the putative *Cpr100A* enhancer (Fig 2D), but not in the *Plc21C* enhancer (Fig 2E). Thus, only the former behaved as a direct Hh target; the *Plc21C* enhancer is responsive to Hh pathway activation, but this activity does not depend upon the Ci/Gli binding sites. Thus, altogether, in addition to the top scoring, previously validated proximal *ptc* enhancer, three of the 16 novel predicted enhancers were validated by the chicken *in ovo* electroporation assay, for an overall success rate of 4/17 or 23%.

We next tested whether additional information would further improve prediction of Hh enhancers. We searched the list of clusters in S4 Table for regions annotated to genes that are known or likely Hh targets or that participate in Hh-regulated developmental events, and chose regions linked to *roadkill* (*rdx*), *retinal homeobox* (*Rx*), *gooseberry* (*gsb*) [62–64], and two additional regions of the *patched* gene (*ptc* ^*-2*.*8*^ and *ptc* ^*+5*.*3*^) for testing. Of these five cluster regions, only *rdx* tested positive in the chicken neural tube assay (Fig 2F), reflecting a similar 20% success rate.

To learn more about the sensitivity of the chicken neural tube assay, we also tested 18 clusters with Ci/Gli sites of low MSS (0.75–0.8). These may represent clusters of sites of low affinity Ci/Gli binding. The regions tested included the known enhancers regulating the *wingless* (*wg)* and *decapentaplegic* (*dpp)* loci (S5 Table). However, none of these showed activity in the chicken neural tube.

**Fig 3:**
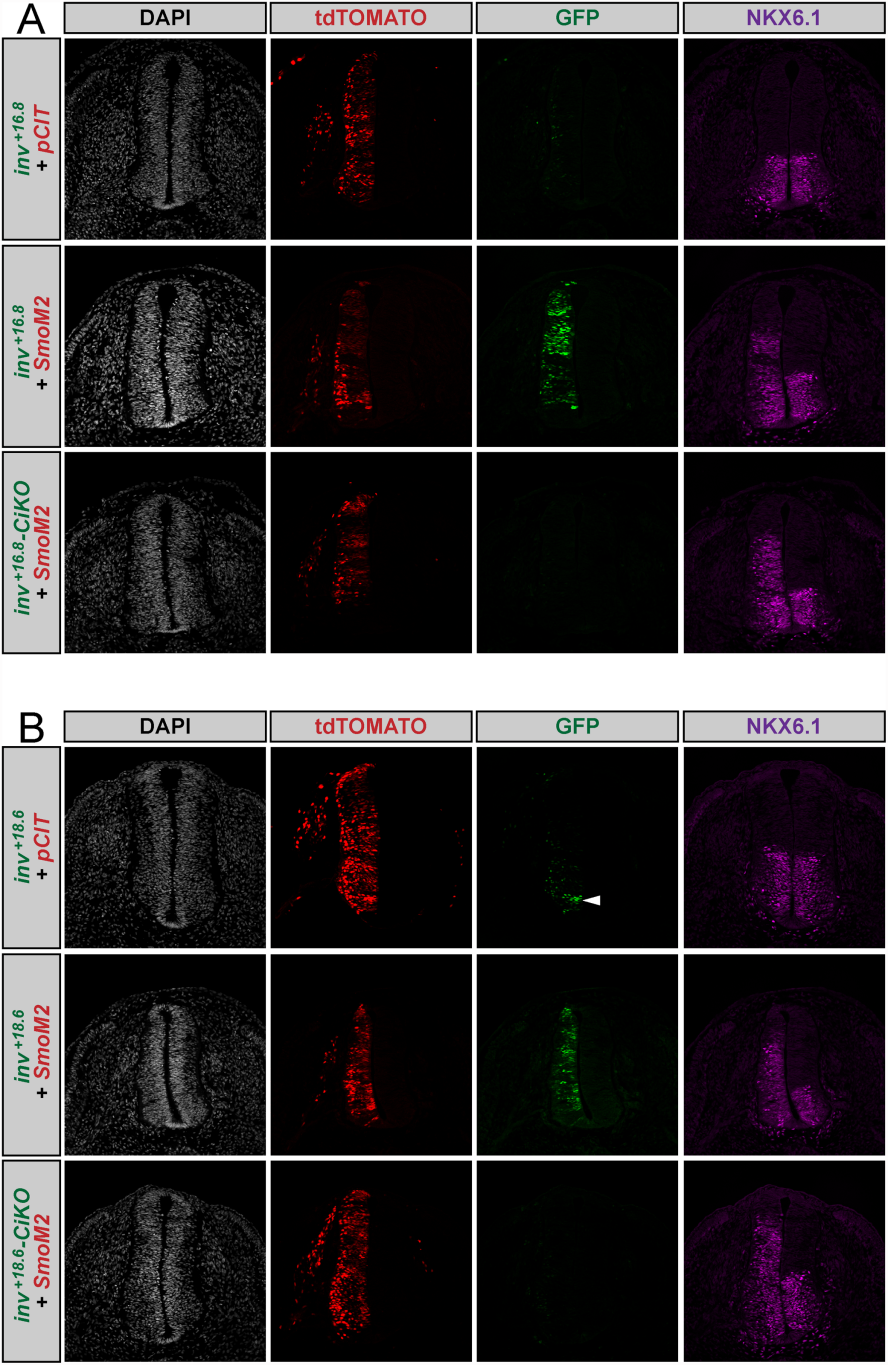
Endogenous expression of *inv* ^*+16*.*8*^ and *inv* ^*+18*.*6*^ in the chicken neural tube. Transverse sections of Hamburger-Hamilton stage 21–22 chicken embryos are shown. DAPI (grayscale, far left column) depicts nuclei. tdTOMATO (red, middle left column) marks cells electroporated with pCIT. GFP (green, middle right column) reports enhancer activation. Anti-NKX6.1 antibody staining (magenta, far right column) denotes Hh-responsive cells. (A) Chicken embryos electroporated with *inv* ^*+16*.*8*^ show no GFP expression in the chicken neural tube. (B) Chicken embryos electroporated with *inv* ^*+18*.*6*^ exhibit GFP expression (white arrowhead).

Having identified two closely associated novel regions of the *inv* gene that both act as Hh enhancers in the chicken electroporation assay (Fig 2B and 2C), we next utilized this assay to further examine these regions. While both enhancers respond to SmoM2 co-electroporation, only one (*inv* ^*+18*.*6*^), drives EGFP expression in response to endogenous levels of Hh signaling (i.e. in the absence of *SmoM2* co-electroporation) (Fig 3).

**Fig 4:**
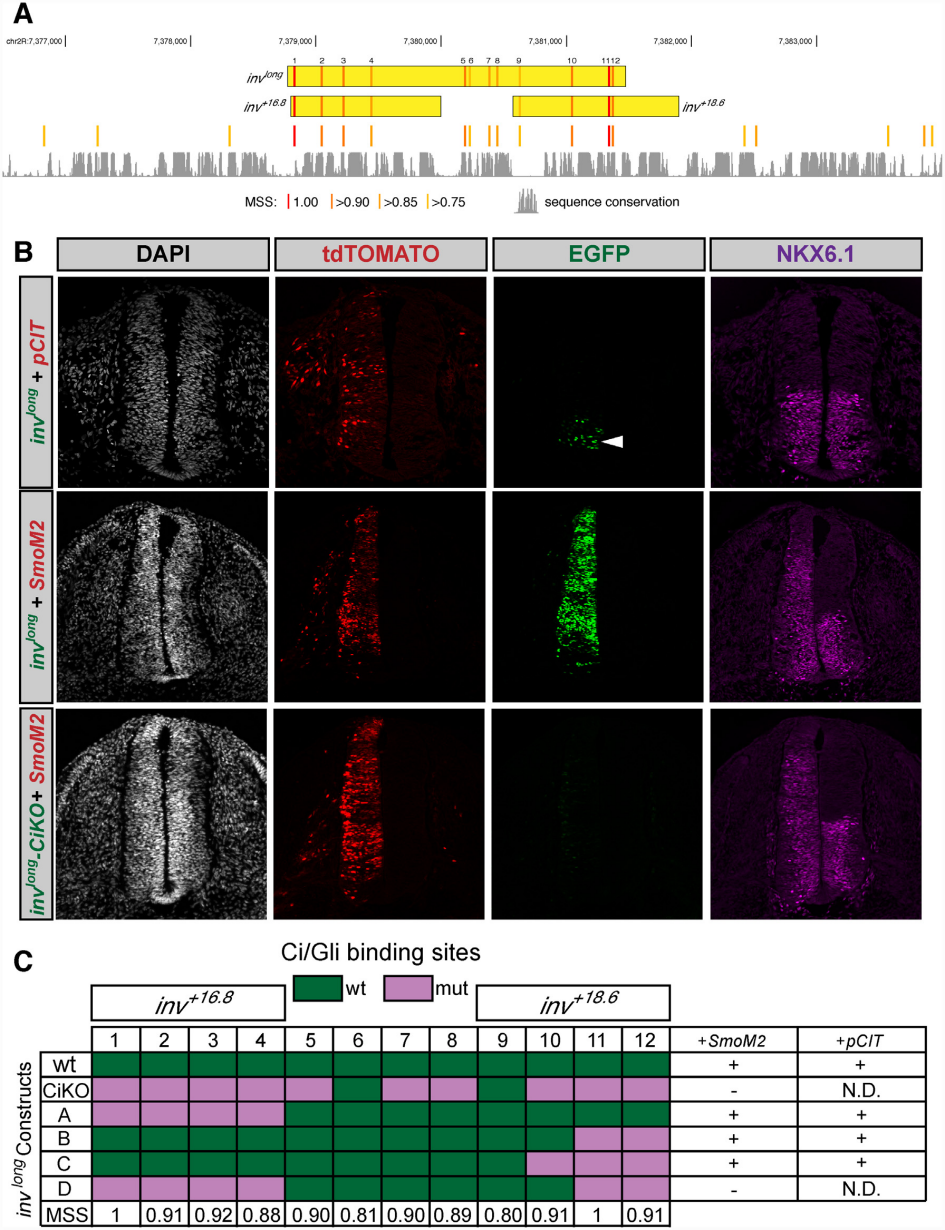
Expression of a complex *inv* enhancer in the chicken neural tube and *Drosophila* wing imaginal disc. (A) Genomic landscape of the *inv* locus depicting the *inv* ^*long*^, *inv* ^*+16*.*8*^ and *inv* ^*+18*.*6*^ constructs. Ci/Gli binding sites are shown as red/orange bars; the intensity of red coloration indicates the MSS. Sequence conservation is indicated by the track at bottom of the panel. (B) Transverse sections of Hamburger-Hamilton stage 21–22 chicken embryos are shown as in Fig 5. DAPI (gray, far left column) depicts nuclei. tdTOMATO (red, middle left column) marks cells electroporated with pCIT or *SmoM2*. GFP (green, middle right column) reports enhancer activation. Anti-NKX6.1 antibody staining (magenta, far right column) marks Hh-responsive cells. The *inv* ^*long*^ (top row) enhancer demonstrates GFP expression in the ventral neural tube (white arrowhead). The expression of *inv* ^*long*^ is strengthened and broadened with co-electroporation of *SmoM2* (middle row). Mutagenesis of Ci/Gli binding sites demonstrates that enhancer activity is Ci/Gli dependent (bottom row). (C) Tabulation of activity in the chicken neural tube of *inv* ^*long*^ constructs containing different Ci/Gli site compositions. Green boxes indicate wild type Ci/Gli sequences; purple boxes indicate mutated Ci/Gli sites. Constructs that have functional Ci/Gli sites that correspond to *inv* ^*+18*.*6*^ (Construct A) or *inv* ^*+16*.*8*^ (Construct B and C) exhibit GFP expression in the neural tube. However, the central Ci/Gli binding sites are insufficient to drive enhancer activity alone (construct D).

Notably, though it is not in the top 16 predictions, S4 Table lists a third cluster in this region of the *inv* locus, lying between the two active regions tested above. Thus, we also tested a fragment spanning all three of these predicted *inv* Ci/Gli clusters, containing a total of 12 Ci/Gli binding sites (*inv* ^*long*^) (Fig 4A). This larger construct is activated both by endogenous SHH in the ventral neural tube and by co-electroporation of *SmoM2* (Fig 4B). Furthermore, a construct (*inv* ^long^-CiKO) containing mutations in 10 of the 12 Ci/Gli binding sites identified computationally (only the two Ci/Gli sites with lowest MSS were left intact) fails to activate EGFP expression, even when co-expressed with *SmoM2* (Fig 4C), confirming the Hh-dependent activity of this large complex enhancer. Further selective mutagenesis of Ci/Gli sites within the larger fragment demonstrates that, in the absence of the *inv* ^*+16*.*8*^ and *inv* ^*+18*.*6*^ Ci/Gli clusters, the central cluster of Ci/Gli binding sites is unable to drive enhancer activity in the chicken neural tube (Construct D, Fig 4C).

### Functional verification of Ci-driven enhancers in transgenic *Drosophila*

**Fig 5:**
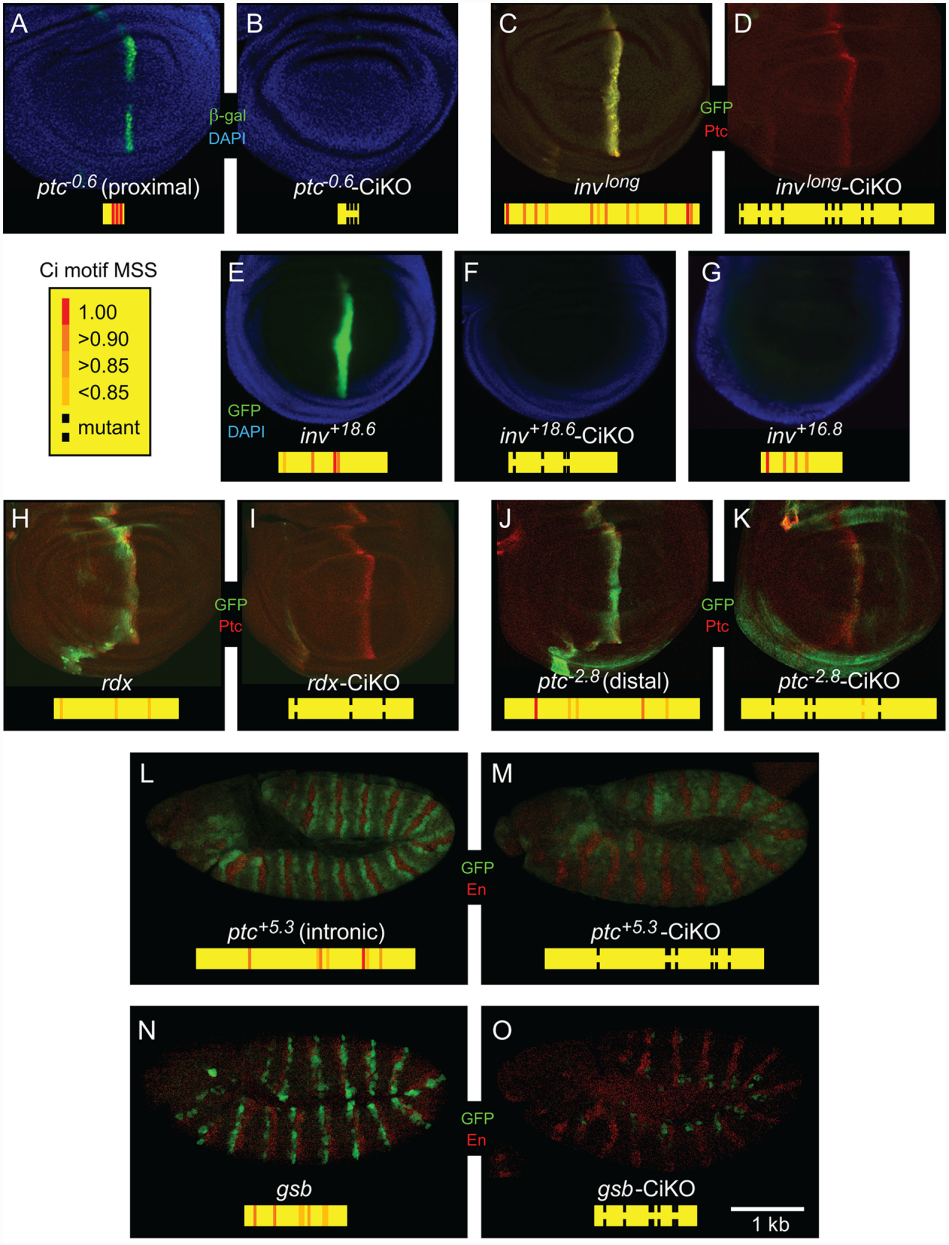
Novel enhancers directly respond to Hh signaling in the wing imaginal disc and embryo. (A-K) β-galactosidase or GFP marks the expression of enhancers in the pouch of the wing imaginal disc. A diagram of the fragments tested and location and MSS for all Ci/Gli sites is shown for each candidate (yellow rectangles). Each wild type enhancer responds to Hh signaling along the anterior-posterior compartment boundary of the wing disc, with the exception of *inv* ^*+16*.*8*^(G). Active enhancers lose Hh responsiveness in the wing imaginal disc when predicted Ci/Gli binding sites are mutated, as shown in the right of each panel. (L-O) GFP marks the expression of the noted enhancers in the embryo. *En* expression (red) marks cells producing Hh ligand. When the predicted Ci/Gli binding sites in these enhancers are mutated (M-O), activity in Hh-responsive cells is severely reduced.

To further verify enhancer function in *Drosophila*, we generated transgenic reporter flies in which EGFP was driven by predicted enhancers and examined gene expression in two of the best-studied Hh-responsive contexts: the stage 9–13 embryo (when Hh signaling is active during development of a variety of tissues) and the anterior/posterior boundary of the larval wing imaginal disc [21,22,26]. The top computational hit, upstream of the *ptc* gene (Table 1, Fig 5A) has three consensus Ci/Gli binding sites and was previously shown to harbor enhancer activity [21]. This conserved cluster was examined as a minimal fragment, (*ptc* ^*-0*.*6*^), which was able to respond to Hh signaling in the wing (Fig 5A). When the three consensus Ci/Gli binding sites were mutated, enhancer activity was abrogated (Fig 5B), confirming that enhancer activity directly depends upon function of the Ci/Gli binding motifs. This region was also found to have enhancer activity in a recent unbiased search for imaginal disc enhancers [65].

We next examined the other novel 16 top computationally predicted enhancers in *Drosophila* and found that three regions exhibited enhancer activity in the fly assay. Although *inv* ^*+16*.*8*^ and *inv* ^*+18*.*6*^ were both active in the chicken neural tube assay when co-electroporated with *SmoM2* (Fig 2B and 2C), only *inv* ^*+18*.*6*^ responded in the wing imaginal disc (Fig 5E–5G). This *inv* ^*+18*.*6*^ enhancer was also the only enhancer to demonstrate positive activity in the chicken ventral neural tube in the absence of *SmoM2*, in response to endogenous Hh expression (Fig 3). When the four predicted Ci/Gli binding sites with higher MSS were mutated in *inv* ^*+18*.*6*^, this enhancer was no longer able to respond to Hh signaling in the wing imaginal disc (Fig 5F), demonstrating its Hh-dependent activity. A larger construct (*inv* ^*long*^), encompassing the four Ci/Gli sites in *inv* ^*+16*.*8*^, the four in *inv* ^*+18*.*6*^, and the intervening cluster of four predicted sites that was tested in the chicken assay in Fig 4A, was also able to drive expression in Hh-responsive cells of the wing imaginal discs of transgenic flies (Fig 5C). The *in vivo* activity of this genomic fragment depended on the predicted Ci/Gli sites (Fig 5D), confirming it as a direct Ci/Gli target enhancer.

In addition to confirming direct Hh-responsiveness of the *ptc* and *inv* enhancers, we also examined the other predicted enhancers in Table 1 in transgenic fly assays. Both *hth* and *Plc21C* showed enhancer activity in the transgenic fly assay, but neither was Hh-dependent (S4 Fig). *Hth* exhibited a segmented expression pattern in the fly embryo, which remained unaltered after mutagenesis of the Ci/Gli binding sites (S3A and S3B Fig). *Plc21C* was expressed in the fly gut and expression persisted after mutation of the Ci/Gli binding sites (S3C and S3D Fig), consistent with the results in the chicken neural tube assay (Fig 2E).

Examination of the five additional Ci/Gli clusters selected from known or suspected Hh target genes yielded four potential Hh-responsive enhancers: *rdx*, *ptc* ^*-2*.*8*^, *ptc* ^*+5*.*3*^ and *gsb*. A Ci/Gli cluster in the intron of *roadkill* (*rdx)* was active at the A/P boundary of the wing imaginal disc in Hh-responsive cells (Fig 5H). Mutating the predicted Ci/Gli sites within this cluster abrogated its activity (Fig 5I). *Rdx* had previously been shown to be genetically downstream of Hh signaling [62], but the enhancer that mediates this response had not been identified. The *Rdx* enhancer identified here also responds to Hh in the chicken neural tube assay (Fig 2F).

Within the *ptc* locus, two other Ci/Gli binding site clusters are computationally predicted in addition to the previously identified promoter-proximal enhancer that topped the list. The first of these, *ptc* ^*-2*.*8*^, is found 2.8 kb upstream of *ptc*, and contains 5 predicted Ci/Gli binding sites. When examined in the wing imaginal disc, *ptc* ^*-2*.*8*^ responds with a stripe of expression largely overlapping Ptc positive cells (Fig 5J). Upon mutation of the predicted Ci/Gli binding sites in this novel enhancer, its ability to respond to Hh is greatly reduced (Fig 5K). A second cluster of Ci/Gli sites in the first intron of *ptc* (*ptc* ^+5.3^) is also predicted. This putative enhancer contains 7 predicted Ci/Gli binding sites, one of which matches the optimal consensus site recognized by Ci/Gli. In flies containing this transgene, *ptc*-like reporter gene expression is seen in the embryo (Fig 5L), but not the wing disc (data not shown). Two stripes of enhancer expression are detected, proximal to cells secreting Hh ligand, marked by *En*, in all segments of the embryonic ectoderm. After mutation of the predicted Ci/Gli binding sites contained within this enhancer, the segmentally repeated stripes are lost (Fig 5M).

Finally, a region with several clusters of Ci/Gli binding sites was identified downstream of the *gooseberry (gsb)* coding region. *Gooseberry*, a segment polarity gene, is part of the Hh-Wnt segmentation network, but no direct Ci/Gli target enhancer has been identified [64]. The only known enhancer of *gsb*, which does not appear to be regulated by Ci/Gli, is 5' of the gene [66]. The 3' enhancer identified by our analysis contains five predicted Ci/Gli binding sites and is active in segmental stripes in the embryonic ectoderm of transgenic *Drosophila*, posterior to each stripe of Hh-secreting cells at stage 11 (Fig 5N). Upon mutation of the Ci/Gli binding sites, activity is attenuated, suggesting that the *gsb* enhancer requires direct Ci/Gli input in order to respond to Hh signaling in the embryo (Fig 5O).

**Fig 6:**
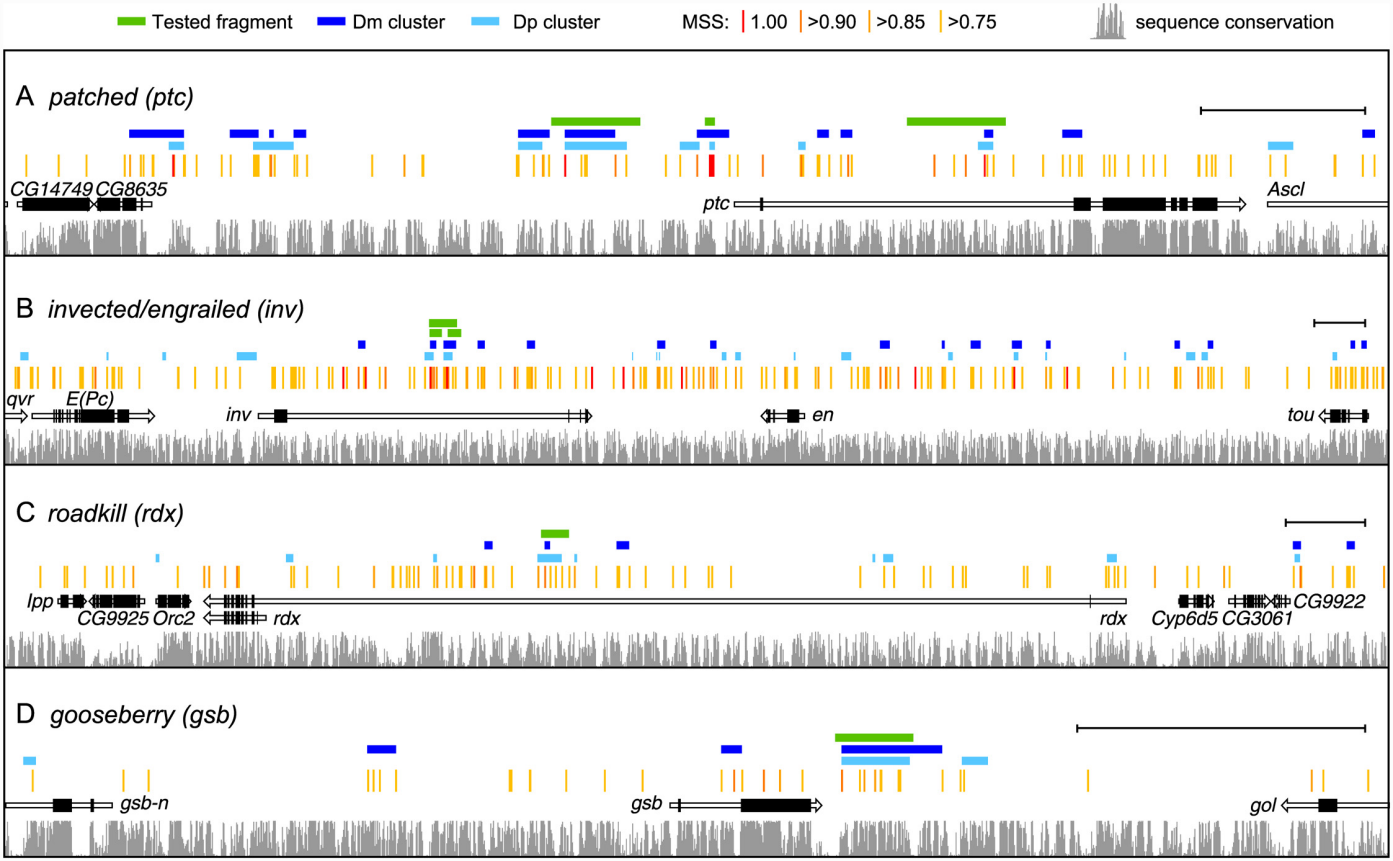
Mapping six Hh regulated enhancers in four genetic loci. (A-D) Genomic landscape of the *ptc*, *inv*, *rdx* and *gsb* loci with fragments tested marked by green bars. All predicted Ci/Gli binding sites are highlighted (red/orange tick marks, annotated according to MSS, as noted at top of Fig). The sequence conservation track (gray bars) marks conservation among the 12 sequenced *Drosophila* species, whereas the dark and light blue bars represent clusters of predicted Ci/Gli binding sites in Dm and Dp, respectively. Black brackets at right indicate 5Kb.

Overall, the fly assay functionally verified six Hh-dependent enhancers out of 22 tested, for a success rate of 27%. The genomic locations of these enhancers, relative to the gene locus, are presented in Fig 6. One additional enhancer, *Cpr100A*, was demonstrated to be Hh-dependent in the chicken, but had no activity in the fly assay; thus, it must be considered a potential regulatory element. This result suggested that *Cpr100A* might have been a false-negative in the fly assay, and prompted us to examine it, along with all of the other predicted enhancers, in a third site of Hh signaling, the adult testis. Although the testis depends on Hh signaling, none of the predicted enhancers were active in this tissue. It is possible, however, that the *Cpr100A* cluster (or any other predicted enhancer that is negative in the chicken and/or fly assays) may be active in another tissue that was not examined [67]. Altogether, both assays established 7/22 (31.8%) of tested Ci/Gli clusters as Hh enhancers, six of which are novel (the potential *Cpr100A* element is not included in this count).

## Discussion

Homotypic clustering of transcription factor binding sites has been observed in multiple settings and has been successfully used to identify potential enhancers [28,29,30,31,68]. Since all but one of the known *Drosophila* Hh-driven enhancers contain two or more Ci/Gli sites, we assessed the extent to which clustering of Ci/Gli sites can be used to predict the location of Hh-dependent enhancers, a question that has not previously been directly tested. To do this, we utilized a background correction method that preserves local nucleotide topography to allow us to identified genomic regions that appear to have unusually dense Ci/Gli binding site representation and tested the extent to which these regions can function as Hh-dependent enhancers.

To establish background genomes for comparison of Gli density, we used a strategy that randomly flips each base to its complimentary partner. This approach maintains the GC/AT landscape of the native *Drosophila* chromosomes. Overall, only 43% of the *D*. *melanogaster* contains G or C bases while the consensus Ci/Gli binding site itself is 67% GC rich [14,69]. Distribution of GC content has been strongly correlated with gene density and other genomic features and the importance of maintaining the original properties of the native sequence when generating a background comparison has been discussed previously [70]. Other background generation methods that preserve dinucleotide frequencies also exist [70,71]. Additional comparisons would be needed to determine which background strategy best strengthens enhancer detection.

The success rate of functional enhancer identification based on the approach used here was 23%, suggesting that clustering of Ci/Gli sites alone is not sufficient to effectively predict Hh-regulated enhancers. However, this success rate increased to 80% when examining Ci/Gli clusters associated with known or suspected Hh target genes. Together, these data indicate that Ci/Gli clustering is not, by itself, an effective means to predict Hh-regulated enhancers. While some Hh enhancers can be identified by virtue of Ci/Gli homotypic clustering, not all homotypic clusters function as enhancers. Since one of the previously identified Hh enhancers (in orthodenticle) only has one Ci/Gli site [22], it is also clear that the presence of clustered Ci/Gli sites is not a requirement for functional Hh enhancers. However, in the context of additional information, clustering can be used as one criterion to predict enhancers within a suspected Hh target gene locus. Future studies will be necessary to determine whether the presence of multiple Ci/Gli sites are more effective predictors of Hh-regulated enhancers associated with putative Hh target genes, or whether a single Ci/Gli site is equally likely to drive Hh-dependent target gene expression.

Given that Ci/Gli binding site clustering alone is not sufficient to identify Hh-regulated enhancers, this raises the question: what is an effective method to identify Hh-regulated enhancers? One possibility is to pair Ci/Gli binding sites with sites for other transcriptional co-activators or co-repressors. *De novo* motif analysis has been performed previously as part of ChIP-chip analysis of GLI repressor binding in the developing limb [18]. More recent studies suggest that GLI proteins cooperate with SOXB1 proteins to drive Hh-regulated gene expression during spinal cord development [20,55]. However, specific co-factor identification may yield only tissue-specific Hh-regulated enhancers. Thus, other approaches include: 1) examining Ci/Gli binding site association with active or repressive chromatin modifications, which has been recently used to investigate Hh-regulated enhancers in the developing neural tube [72], and 2) investigating Ci/Gli binding site location near sites of open chromatin using techniques such as DNAse I hypersensitivity and FAIRE [73,74]. It is likely that a combination of these methods will be required to effectively identify a more complete set of Hh-regulated enhancers on a genome wide basis.

One intriguing finding from this work is the identification of multiple discrepancies between the chicken neural tube and transgenic fly assays (Table 1). These data emphasize the importance of testing putative enhancers in diverse assay systems to provide several different contexts in which an enhancer can show activity. The chicken neural tube assay is a quick and inexpensive strategy that, in a large-scale study, could improve throughput. It has been successfully used previously to identify Hh-regulated mouse enhancers [20,55], and is used here to validate Hh-regulated fly enhancers. However, because some enhancers may require additional species-specific information that is not present in the chicken neural tube, false negative calls are a limitation of this assay. Further, the requirement for context-specific information may also restrict the utility of this assay in the identification of general Hh-regulated enhancers [75]. Along these lines, analysis of 18 clusters containing Ci/Gli sites of lower predicted affinity, including the known Hh enhancers in the *wg* and *dpp* loci [24,26], showed no activity in the chicken neural tube (S5 Table). Thus, this assay may only detect Hh enhancers with high affinity Ci/Gli binding sites, thereby missing some true positives [22]. Nevertheless, the assay can be useful to dissect enhancer activity in the context of a complex developing tissue (Fig 4).

The computational study presented here can be compared with a recent analysis of potential Ci/Gli-driven enhancers in *Drosophila*, by Biehs et al., who fused Ci^ACT^ (activator) and Ci^REP^ (repressor) proteins with DNA adenine methyltransferase (Dam) domains to define chromatin regions in stage 10–11 embryos that are occupied by Ci/Gli *in vivo* [16]. That study listed 1743 sites bound by Dam-Ci fusion proteins; of these, 55 sites (3%, listed in S6 Table) were represented in clusters that were selected by our computational analysis. This limited overlap is likely due to two factors. First, since the computational study was limited to analysis of larger clusters, enhancers that are driven by one or two Ci/Gli sites were not selected, by design. Second, because the DamID study was performed in 2–6 hour embryos, Ci/Gli binding events were likely limited to chromatin regions that were accessible at that developmental stage. Of the seven previously known Hh/Gli-regulated enhancers, the DamID approach identified Ci/Gli binding to two (*ptc* and *wg*), while the computational strategy described here detected three (*ptc*, *wg* and *knot*). The other four previously known enhancers (*stripe*, *hairy*, *dpp* and *orthodenticle*) were not detected computationally because those enhancers have only two Ci/Gli sites (our filters selected clusters of 3–10). Of the new enhancers functionally confirmed in our study, none were found to harbor protected regions in the DamID assay. Biehs et al. used expression assays to identify 147 genes whose expression appeared to correlate with Hh signaling activity. They then asked, of these 147 genes, how many had protected regions within or adjacent to the transcription unit? Protected regions were identified as DamAct or DamRep protection and consisted of a total 2108 protected regions. They identified 52 genomic regions that were DamID-protected and showed expression changes when Hh signaling was modulated. Thus, 35% of the genes that appear to be targets (as assessed by their expression modulation) showed some DamID protection, but only 2.5% of the total DamID protected regions were found to be probable Hh targets [16]. Four of these 52—but none of the validated enhancers—can be found in the list of 55 sites common to the two studies.

An important aspect of the present study is that the direct Hh dependency of all enhancers was verified by Ci/Gli binding site mutagenesis. While expression assays such as those used by Biehs et al. clearly demonstrate a Hh response, they do not establish whether this response is direct or indirect and do not confirm that the response is mediated through the Ci/Gli binding sites in the candidate enhancers. Indeed, of the top 17 clusters detected computationally, we found four direct targets and two additional enhancers that showed apparent expression in *ptc*-expressing cells, but this expression persisted after mutation of the Ci/Gli sites (S4 Fig) suggesting that other factors might be responsible for this enhancer activity. This raises a cautionary note about assigning potential Hh, or any signaling cascade, responsiveness in the absence of functional verification [76].

Using homotypic Ci/Gli site clustering as a criterion together with functional analyses, we have doubled the number of previously verified *Drosophila* Ci/Gli-dependent enhancers, including multiple distinct enhancers that regulate a single Hh-responsive gene (i.e., *ptc*, *inv*, and *gsb*). Further testing of other candidate clusters identified in this study might further enlarge the pool of known Hh-responsive enhancers that are active in diverse tissues and organs, providing a robust substrate for the future dissection of the rules that underlie context-specific enhancer function.

## Supporting Information

S1 Fig Assessment of GC content surrounding Ci/Gli sites in the *Drosophila melanogaster* genome. Proportion of sequence that is GC in the 50 bp surrounding predicted Ci/Gli sites on each Dm chromosome is displayed in the left panel. Chromosomes are color-coded: 2L (red); 2R (light blue); 3L (blue); 3R (green); 4 (yellow), and X (purple). Notably, all *Drosophila* chromosomes have similar distributions of GC content surrounding the predicted existing Ci sites, except for Chromosome 4, which is considerably more AT rich in regions surrounding predicted Ci sites (yellow line). Three different models (Flip GC/AT, Shuffle 3-mer and Random, see Materials and Methods for details) were used to create three background sequences for each chromosome and the GC content in 50 bp surrounding each Ci/Gli site was compared among the models. Black is used for the randomized model since all chromosomes collapse on the same distribution. Error bars show standard error of the mean for the 100 chromosomes in each model. Only the Flip GC/AT model recapitulates the GC profile of the native Dm genome.
(PDF)

S2 Fig Construction of background genomes and determination of cluster enrichment. (A) The actual number of predicted Ci/Gli sites (≥0.75 MSS) determined in each Dm chromosome is shown by the green lines. The Flip GC/AT method was used to create 1000 background sequences and the number of predicted Ci/Gli sites was tallied for each sequence. Box plots show that randomized chromosomes contain substantially fewer predicted Ci/Gli sites. Brackets represent the range in total number of Ci/Gli sites across the background sequences for each chromosome. (B) To correct for the depleted number of predicted Ci/Gli sites and create background chromosomes that would closely mirror the native Dm genome, the location (coordinates) and type (sequence) of all predicted Ci/Gli sites in each of the 1000 background sequences were recorded and pooled. Background genomes were then constructed by randomly selecting coordinates from the pools so that the composition (number and site type) matched that of the corresponding Dm chromosome. (C) Enrichment of clusters of 3–10 Ci/Gli sites relative to the background chromosomes was then determined. The example shows analysis of enrichment for clusters of 3 Ci/Gli sites (blue boxes). The Dm chromosome (black line) is compared with 100 background chromosomes (grey lines); the diagram shows only three of the 100 background chromosomes. In a moving window, each group of three Ci/Gli sites was delineated in the Dm chromosome (one such cluster is outlined in orange) and the average number of Ci/Gli sites was determined within that same genomic space in each of the 100 background chromosomes. The cluster outlined by the orange box is considered enriched if the average number of sites in the Dm chromosome is ≥4 fold more than the average number of Ci/Gli sites per background chromosome.
(PDF)

S3 Fig Expression of *hth* and *Plc21C* regions in the fly are not Ci/Gli-dependent. Both *hth* and *Plc21C* drive GFP expression in the fly embryo. *Hth* exhibits expression in the brain as well as a punctate segmental pattern parallel but outside of En expression (shown in red) which marks cells that produce and secrete Hh ligand (A,B). *Plc21C* expresses throughout the gut (C). Expression for both constructs is not Hh dependent since it persists after mutation of Ci/Gli binding sites (B and D).
(PDF)

S1 Table 9-mers with a minimum level (≥0.75) Ci matrix similarity score. (XLSX)

S2 Table PCR primers used to amplify genomic DNA from the *D*. *melanogaster* genome (build dm3). (XLSX)

S3 Table Distribution of predicted Ci/Gli sites across chromosomes. (XLSX)

S4 Table Predicted clusters for the *Drosophila melanogaster* genome (dm3). Columns A-Q are labeled accordingly in row 1. Columns R through AC represent sequence for each *Drosophila* species that corresponds to the multiple sequence alignment (9-mer at the position of the Ci/Gli site in Dm). Number of species that show 100% conservation is shown in column AD. The number of sites assigned to each locus is listed in column AE (boundaries between loci are considered as half of the distance between two neighboring loci). Column AF indicates the number of sites in the locus with MSS≥0.81.
(XLSX)

S5 Table Clusters containing Ci/Gli sites of low MSS tested in the chicken neural tube assay. (XLSX)

S6 Table Overlap between clusters predicted in this study and DamID protected sites. Asterisks indicate four sites that map to one of the 52 probable Ci target genes identified by Biehs *et al*. [16].
(XLSX)
