# Supplementary figures and images for: Identification and Validation of Novel Hedgehog-Responsive Enhancers Predicted by Computational Analysis of Ci/Gli Binding Site Density
Source: PLoS One. 2015 Dec 28;10(12):e0145225. doi: 10.1371/journal.pone.0145225 (PMC4692483; doi:10.1371/journal.pone.0145225)

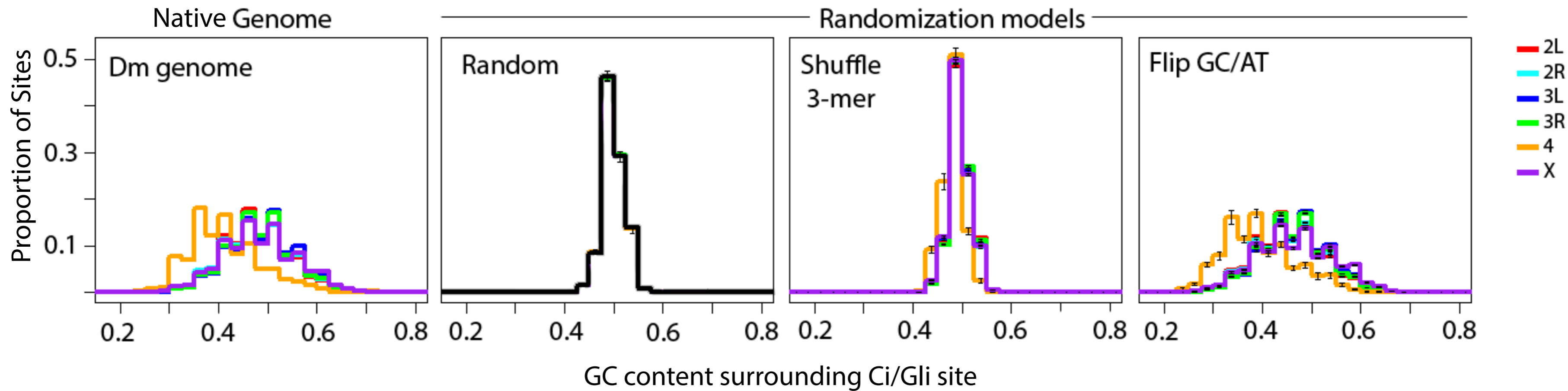

Supplement: S1 Fig — Proportion of sequence that is GC in the 50 bp surrounding predicted Ci/Gli sites on each Dm chromosome is displayed in the left panel. Chromosomes are color-coded: 2L (red); 2R (light blue); 3L (blue); 3R (green); 4 (yellow), and X (purple). Notably, all Drosophila chromosomes have similar distributions of GC content surrounding the predicted existing Ci sites, except for Chromosome 4, which is considerably more AT rich in regions surrounding predicted Ci sites (yellow line). Three different models (Flip GC/AT, Shuffle 3-mer and Random, see Materials and Methods for details) were used to create three background sequences for each chromosome and the GC content in 50 bp surrounding each Ci/Gli site was compared among the models. Black is used for the randomized model since all chromosomes collapse on the same distribution. Error bars show standard error of the mean for the 100 chromosomes in each model. Only the Flip GC/AT model recapitulates the GC profile of the native Dm genome. (PDF) [file pone.0145225.s001.pdf]

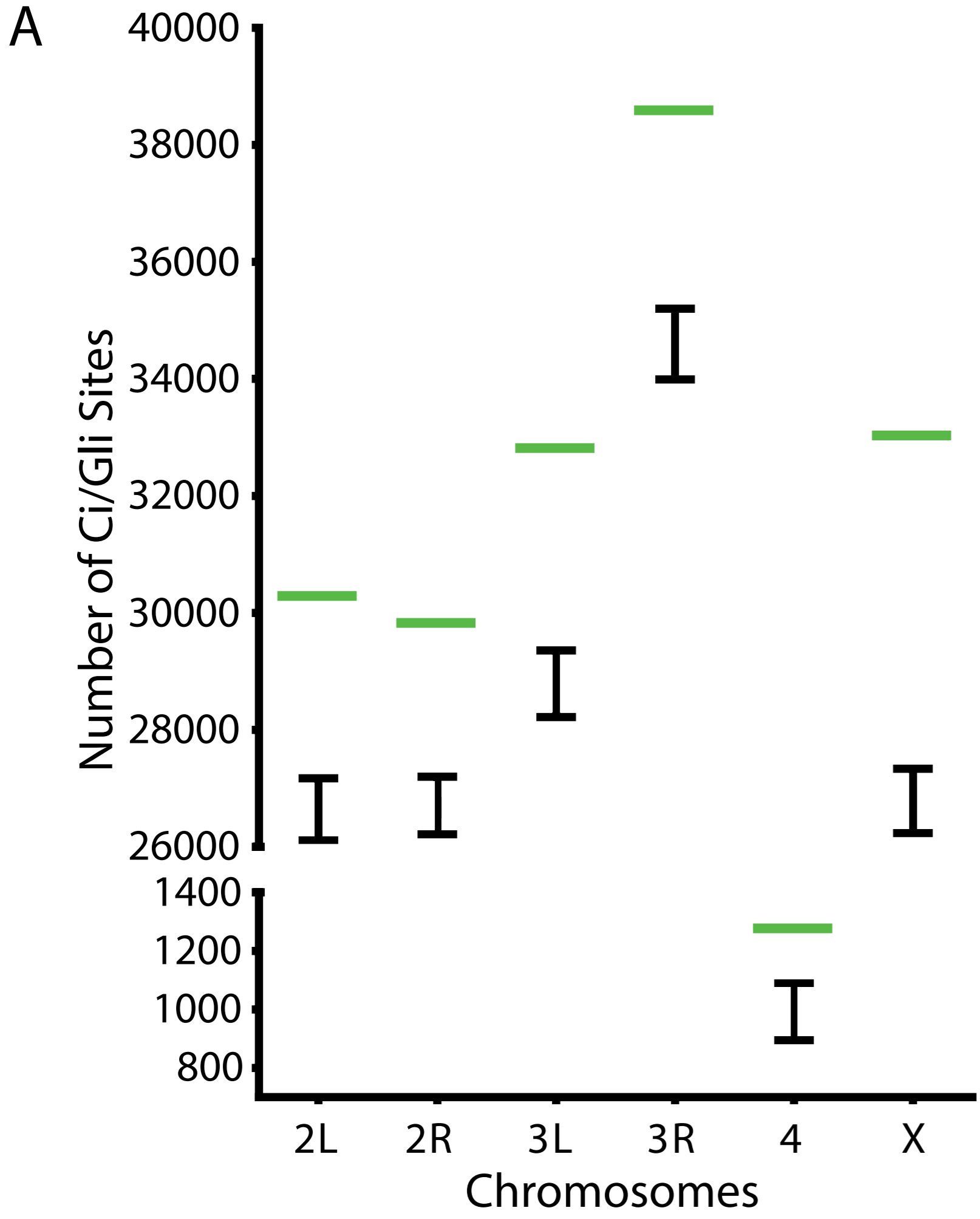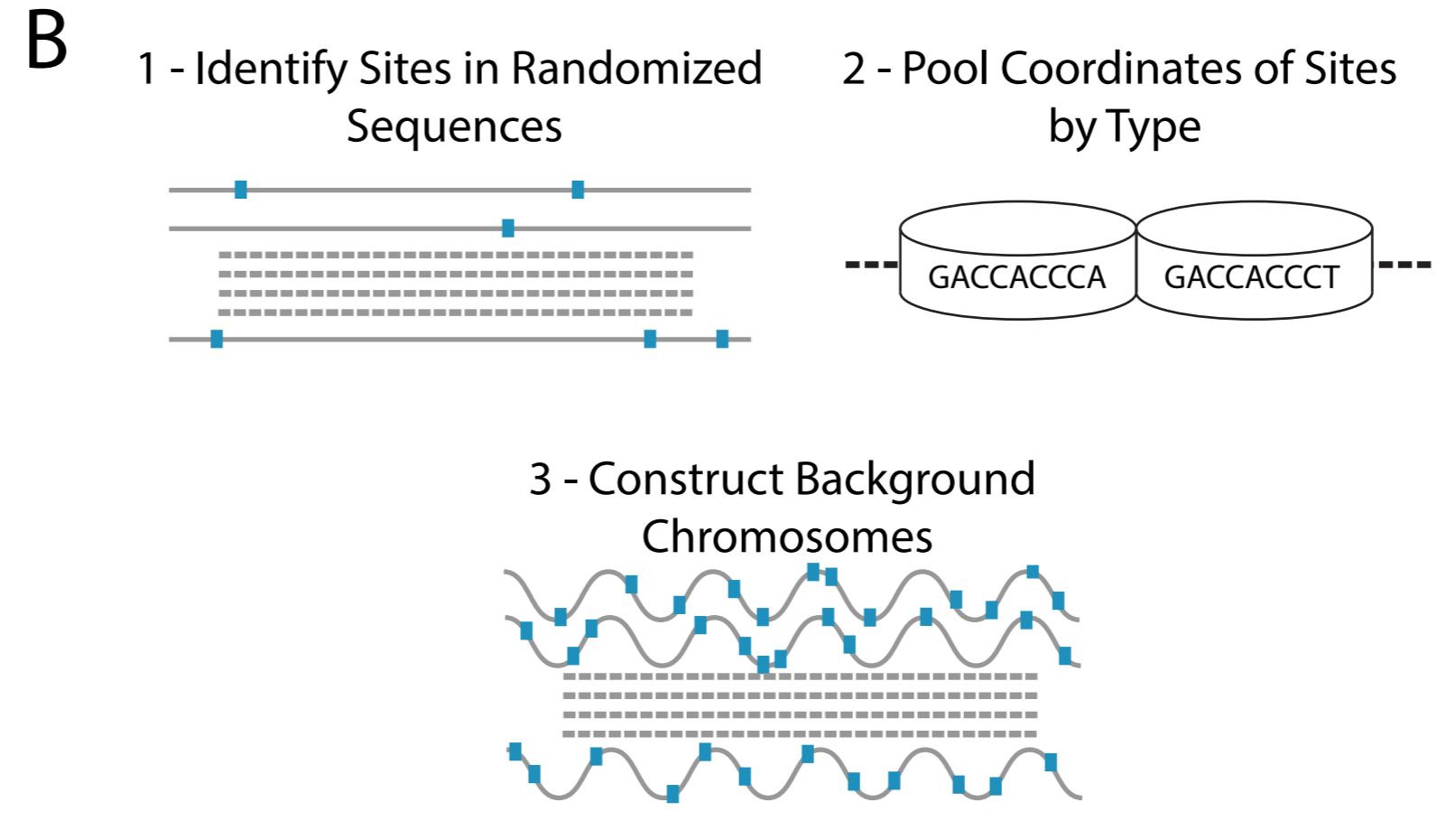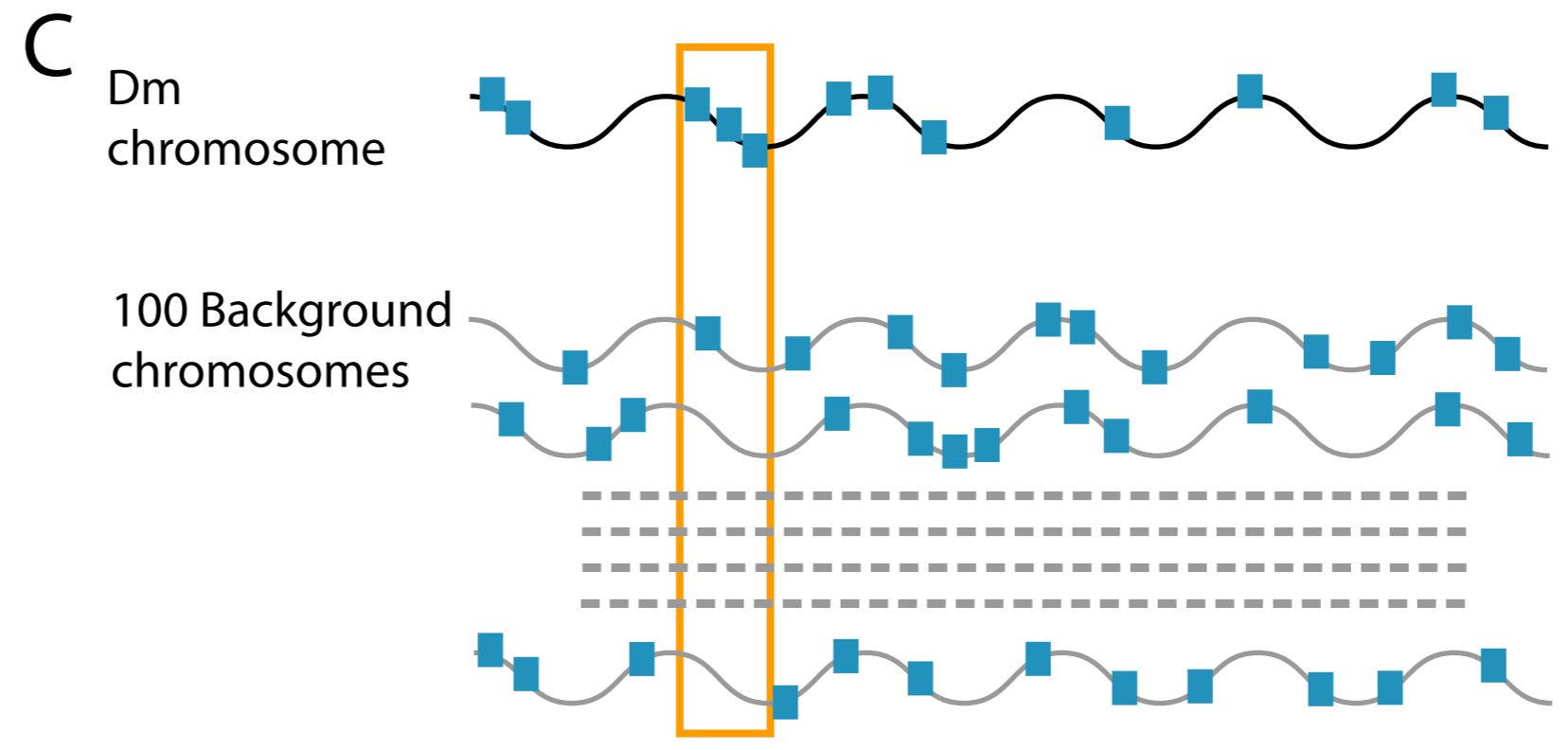

Supplement: S2 Fig — (A) The actual number of predicted Ci/Gli sites (≥0.75 MSS) determined in each Dm chromosome is shown by the green lines. The Flip GC/AT method was used to create 1000 background sequences and the number of predicted Ci/Gli sites was tallied for each sequence. Box plots show that randomized chromosomes contain substantially fewer predicted Ci/Gli sites. Brackets represent the range in total number of Ci/Gli sites across the background sequences for each chromosome. (B) To correct for the depleted number of predicted Ci/Gli sites and create background chromosomes that would closely mirror the native Dm genome, the location (coordinates) and type (sequence) of all predicted Ci/Gli sites in each of the 1000 background sequences were recorded and pooled. Background genomes were then constructed by randomly selecting coordinates from the pools so that the composition (number and site type) matched that of the corresponding Dm chromosome. (C) Enrichment of clusters of 3–10 Ci/Gli sites relative to the background chromosomes was then determined. The example shows analysis of enrichment for clusters of 3 Ci/Gli sites (blue boxes). The Dm chromosome (black line) is compared with 100 background chromosomes (grey lines); the diagram shows only three of the 100 background chromosomes. In a moving window, each group of three Ci/Gli sites was delineated in the Dm chromosome (one such cluster is outlined in orange) and the average number of Ci/Gli sites was determined within that same genomic space in each of the 100 background chromosomes. The cluster outlined by the orange box is considered enriched if the average number of sites in the Dm chromosome is ≥4 fold more than the average number of Ci/Gli sites per background chromosome. (PDF) [file pone.0145225.s002.pdf]

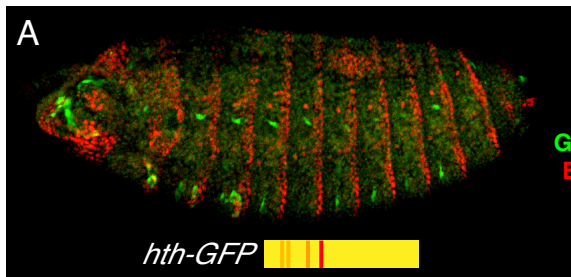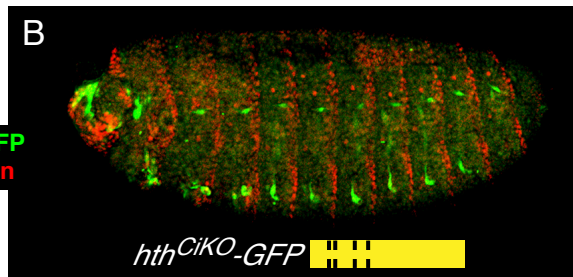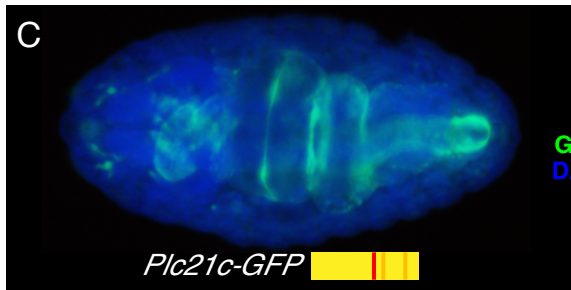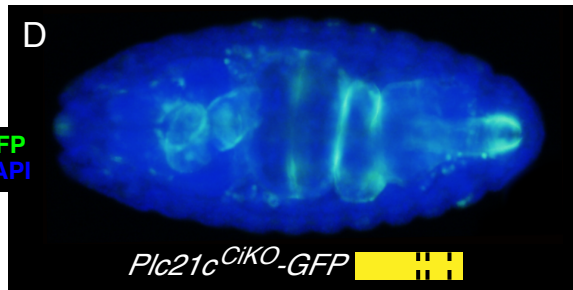

Supplement: S3 Fig — Both hth and Plc21C drive GFP expression in the fly embryo. Hth exhibits expression in the brain as well as a punctate segmental pattern parallel but outside of En expression (shown in red) which marks cells that produce and secrete Hh ligand (A,B). Plc21C expresses throughout the gut (C). Expression for both constructs is not Hh dependent since it persists after mutation of Ci/Gli binding sites (B and D). (PDF) [file pone.0145225.s003.pdf]
